# Prognostic Relevance of Copy Number Losses in Ovarian Cancer

**DOI:** 10.3390/genes15111487

**Published:** 2024-11-19

**Authors:** Andrea Jemma, Alessandra Ardizzoia, Serena Redaelli, Angela Bentivegna, Marialuisa Lavitrano, Donatella Conconi

**Affiliations:** 1 School of Medicine and Surgery, University of Milano-Bicocca, 20900 Monza, Italy; a.jemma@campus.unimib.it (A.J.); a.ardizzoia@campus.unimib.it (A.A.); serena.redaelli@unimib.it (S.R.); angela.bentivegna@unimib.it (A.B.); marialuisa.lavitrano@unimib.it (M.L.); 2 Fondazione Istituto di Oncologia Molecolare ETS (IFOM), The AIRC Institute for Molecular Oncology, 20139 Milan, Italy

**Keywords:** ovarian cancer, biomarkers, copy number loss, cancer stem cells, copy number alterations, numerical aberrations

## Abstract

Background/Objectives: Aneuploidy is a prevalent cancer feature that occurs in many solid tumors. For example, high-grade serous ovarian cancer shows a high level of copy number alterations and genomic rearrangements. This makes genomic variants appealing as diagnostic or prognostic biomarkers, as well as for their easy detection. In this study, we focused on copy number (CN) losses shared by ovarian cancer stem cells (CSCs) to identify chromosomal regions that may be important for CSC features and, in turn, for patients’ prognosis. Methods: Array-CGH and bioinformatic analyses on three CSCs subpopulations were performed. Results: Pathway and gene ontology analyses on genes involved in copy number loss in all CSCs revealed a significant decrease in mRNA surveillance pathway, as well as miRNA-mediated gene silencing. Then, starting from these CN losses, we validated their potential prognostic relevance by analyzing the TCGA cohort. Notably, losses of 4q34.3-q35.2, 8p21.2-p21.1, and 18q12.2-q23 were linked to increased genomic instability. Loss of 18q12.2-q23 was also related to a higher tumor stage and poor prognosis. Finally, specific genes mapping in these regions, such as *PPP2R2A* and *TPGS2A*, emerged as potential biomarkers. Conclusions: Our findings highlight the importance of genomic alterations in ovarian cancer and their impact on tumor progression and patients’ prognosis, offering advance in understanding of the application of numerical aberrations as prognostic ovarian cancer biomarkers.

## 1. Introduction

Advances in high-throughput “omics” technologies produced a huge amount of data, including cancer genomics data. Aneuploidy is a hallmark feature in approximately 90% of solid tumors, reflecting widespread genomic instability [1]. Although its exact role in tumor initiation and progression remains unclear, it is thought to contribute to the complexity of chromosomal aberrations frequently observed in cancer cells. Traditionally, aneuploidy refers to numerical changes involving whole chromosomes, but more recent studies have expanded this concept to include segmental alterations, such as losses or gains of chromosome arms, which are often categorized as copy number alterations (CNAs) [2]. For this reason, it can be challenging to compare different studies because numerical alterations can involve either few genes (CNAs) or entire chromosome region (SCAs—segmental chromosomal aberrations; copy number gains or losses affecting one chromosome arm or a segment within a chromosome arm with at least 100 contiguous oligonucleotide probes) [3].

Numerical aberrations can readily be detected using conventional and molecular cytogenetics techniques, routinely used in clinics, making them appealing biomarkers for patients’ prognosis [2]. As a matter of fact, CNA burden or specific CNAs are associated with poor outcomes in many cancers including neuroblastoma [3,4], breast cancer [5,6], bladder cancer [7], glioblastoma [8,9] and ovarian cancer [10,11,12,13].

Cancer stem cells (CSCs) are a subpopulation of tumor cells known for driving tumor growth, treatment failure, and cancer relapse [14]. In this context, ovarian cancer spheroids, found in malignant ascites, represent that subpopulation, responsible for ovarian cancer dissemination, impairment of treatments effectiveness, and unfavorable patient prognosis [15]. For these reasons, understanding the connection between CNAs and gene expression in CSCs could be vital for revealing how genetic instability contributes to the tumor’s aggressiveness. Moreover, several studies show that a large proportion of genomic changes in cancer cells can be attributed to CNAs, reinforcing their role in driving key aspects of tumor behavior [16].

In a previous work [10], we identified a potential prognostic role of AhRR and PPP1R3C expression in serous ovarian cancer, starting from a detailed bioinformatics analysis of copy number gain arising in CSCs by array comparative genomic hybridization (array-CGH) analysis. In this work, we turn our attention to copy number losses in order to identify in a similar way chromosomal regions or genes with a specific role in ovarian cancer stem cells and, in turn, for patients’ prognosis.

## 2. Materials and Methods

### 2.1. Cell Lines

Ovarian cancer cell lines Caov3, Ovcar5, and Ovcar8 were purchased from ATCC (American Type Culture Collection, Manassas, VA, USA) and Sigma-Aldrich (St. Louis, MO, USA). Caov3 were grown in Dulbecco’s modified Eagle’s medium (DMEM); Ovcar5 and Ovcar8 were grown in RPMI 1640. Both media were completed with the addition of 10% fetal bovine serum (FBS) and 1% penicillin–streptomycin. All reagents were purchased from EuroClone (Milano, Italy). All the cell lines were maintained in a humidified atmosphere at 37 °C with 5% CO_2_.

Ovarian cancer stem cells (represented by ovarian cancer spheroids) were previously isolated and characterized [10]. Briefly, spheroids were generated following an anchorage-independent growth assay starting from the three different cell lines and then characterized by stemness markers’ expression and clonogenic nature.

### 2.2. Array Comparative Genomic Hybridization

Array comparative genomic hybridization (array-CGH) experiments were previously reported [10]. Briefly, DNA was extracted from ovarian CSCs and the corresponding cell lines using an QIAamp DNA Mini Kit according to the manufacturer’s instructions (QIAGEN, Hilden, Germany). After quantification with a Nanodrop ND-2000 spectrophotometer (Thermo Fisher Scientific, Waltham, MA, USA), samples with a concentration over 10 µg/mL and an absorbance ratio A_260/280_ over 1.8 and A_260/230_ over 1.7 were used for analysis. Array-CGH analysis was performed using a SurePrint G3 Human CGH Microarray 8 × 60 K (Agilent Technologies, Santa Clara, CA, USA) according to the manufacturer’s instructions. The arrays were scanned at 2 μm resolution and analyzed using Agilent Feature Extraction and Agilent Cytogenomics v5.2 software (Agilent Technologies, Santa Clara, CA, USA). The Aberration Detection Method 2 (ADM-2) algorithm was used to compute and assist the identification of aberrations in a given sample (threshold = 5.0), assigning a statistical score based on the average quality weighted log ratio (DLRS) of the sample and reference channels. We applied a filtering option of a minimum of three aberrant consecutive probes and a minimum absolute average log2 ratio that differs among all samples and depends on DLRS values. Log2ratio values over 1 identify amplification; values under −1.7 identify complete loss. Log2ratio values over 0.6 identify non-mosaic gains; values under −1 identify non-mosaic losses. Accordingly, log2 ratio values for mosaic gains range between the DLRS value and 0.6 and for mosaic losses between the DLRS value and −1. As a reference genomic DNA sample, woman-matched DNA was provided by Agilent (Agilent Technologies).

### 2.3. Bioinformatic Analyses

#### 2.3.1. Analysis of Genes Involved in Copy Number Losses

The Database for Annotation, Visualization and Integrated Discovery (DAVID, https://david.ncifcrf.gov/tools.jsp, accessed on 29 July 2024) platform was used to identify enriched REACTOME pathways, Kyoto Encyclopedia of Genes and Genomes (KEGG) pathways, and GO (Gene Ontology) terms (BP: biological process, and MF: molecular function) [17]. A *p*-value less than 0.05 was considered statistically significant.

#### 2.3.2. Correlation of Chromosome Aberrations and Clinical Data

To evaluate a potential prognostic role of segmental chromosome aberrations, we analyzed the TCGA-OV (The Cancer Genome Atlas Ovarian Cancer) cohort using the cBioPortal for Cancer Genomics web server (https://www.cbioportal.org/, accessed on 29 July 2024). Ovarian serous cystadenocarcinoma (TCGA PanCancer Atlas) was chosen. Samples with or without loss were selected to form the different groups (4q34.3-q35.2 loss vs. no loss, 8p21.2-p21.1 loss vs. no loss, and 18q12.2-q23 loss vs. no loss), which were compared to identify differences in clinical parameters (survival, clinical, protein, arm-level CNA).

For the cBioportal web server, copy number data from the GDC analysis pipelines were provided in ASCAT format and converted to discrete GISTIC data using the following thresholds: −2 or Deep Deletion indicates a homozygous deletion; −1 or Shallow Deletion indicates a heterozygous deletion; 0 is diploid; 1 or Gain indicates a low-level gain (a few additional copies); 2 or Amplification indicates a high-level amplification (more copies) (https://www.cbioportal.org/).

The Genomic Data Commons (GDC) Data Portal (https://portal.gdc.cancer.gov/, accessed on 29 July 2024) provides access to the subset of TCGA data that has been harmonized against GRCh38 (hg38) using GDC Bioinformatics Pipelines, which provides methods to the standardization of biospecimen and clinical data and the generation of derived data [18]. The CNA pipeline uses either NGS or Affymetrix SNP 6.0 (SNP6) array data to identify genomic regions that are repeated and infer the copy number of these repeats. Three sets of pipelines have been used for CNV inferences: ASCAT, ABSOLUTE, and DNAcopy.

We chose the TCGA-OV project, divided the patients into cohorts according to the tumor stage or age at diagnosis (under or over 63 years), and then we compared the frequency of copy number gain and loss in each cohort.

#### 2.3.3. Analysis of FHOD3, TPGS2, and KIAA1328 Expression in Ovarian Cancer

GEPIA2 (http://gepia2.cancer-pku.cn/, accessed on 29 July 2024) and Kaplan–Meier plotter (https://kmplot.com/analysis/, accessed on 29 July 2024) web servers were used to analyze the correlation between the selected genes’ expressions and patients’ overall survival and disease-free survival in ovarian cancer. The selected cut-off value was “median cut-off” (GEPIA2, OV Dataset) and “auto selected best cut-off” (Kaplan–Meier plotter mRNA Chip). *p*-value < 0.05 was considered statistically significant. OncoDB web server (https://oncodb.org/, accessed on 29 July 2024) was consulted for the analysis of correlation between expression of genes and clinical stage of the tumor. *p*-value < 0.05 was considered statistically significant.

## 3. Results

### 3.1. Lost Genes Were Involved in mRNA Processing

Ovarian cancer spheroids were obtained from three ovarian cancer cell lines (Caov3, Ovcar5, and Ovcar8) belonging to high-grade serous histotype [19,20,21,22,23]. In our previous study, we checked the expression of ovarian cancer stemness markers ALDH, CD44, ABCG2, and NANOG and spheroids’ clonogenic nature through PKH staining [10]. All ovarian cancer spheroids showed an increased expression of stemness markers compared to the corresponding cell line and spheroids’ clonogenic nature. These results validate our model as ovarian cancer stem cells [10].

Firstly, based on the copy number losses shared by all three CSC subpopulations, we identified a list of 1253 genes, which underwent enrichment analyses in GO terms, REACTOME pathways, and KEGG pathways through the DAVID platform (Table S1).

KEGG pathway enrichment analysis revealed a significant decrease in the mRNA surveillance pathway. This pathway serves as a quality control mechanism that identifies and degrades abnormal mRNAs, and it includes nonsense-mediated mRNA decay (NMD), nonstop mRNA decay (NSD), and no-go decay (NGD) [24]. In particular, lost genes involved in this pathway were almost exclusively located on chromosome X, except for the *PPP2R2A* gene located in 8p21.1: *GSPT2* (Xp11.22), *UPF3B* (Xq24), *CSTF2*, *NXF2*, *NXF2B*, *NXF3* (all Xq22.1), *NCBP2L* (Xq22.3), *NXT2* (Xq23), *PABPC1L2A* and *PABPC1L2B* (Xq13.2), and *PABPC5* (Xq21.31). Instead, REACTOME pathway enrichment analysis highlighted a loss of downregulation of SMAD2/3:SMAD4 transcriptional activity.

GO enrichment analysis identified a strong downregulation of gene silencing by miRNA (BP: biological process), with 60 downregulated miRNAs in CSCs, confirmed by a decrease of mRNA binding involved in posttranscriptional gene silencing GO MF (molecular function), with 31 downregulated miRNAs in CSCs.

Interestingly, some GO terms associated with mRNA processing (such as negative regulation of transcription by RNA polymerase II, poly(A)+ mRNA export from nucleus, mRNA 3′-UTR binding, poly(A) binding, and mRNA export from nucleus) were underrepresented in our CSC subpopulations. Moreover, CSCs showed upregulation of angiogenesis, autophagy, G0 to G1 transition, and ERK1 and ERK2 cascade.

Taken together, these results explain some characteristics of our CSCs models and confirm the importance of copy number losses in tumorigenesis and cancer progression.

### 3.2. Copy Number Losses Correlated with Clinical Parameters in the cBioPortal Web Server

**Figure 1:**
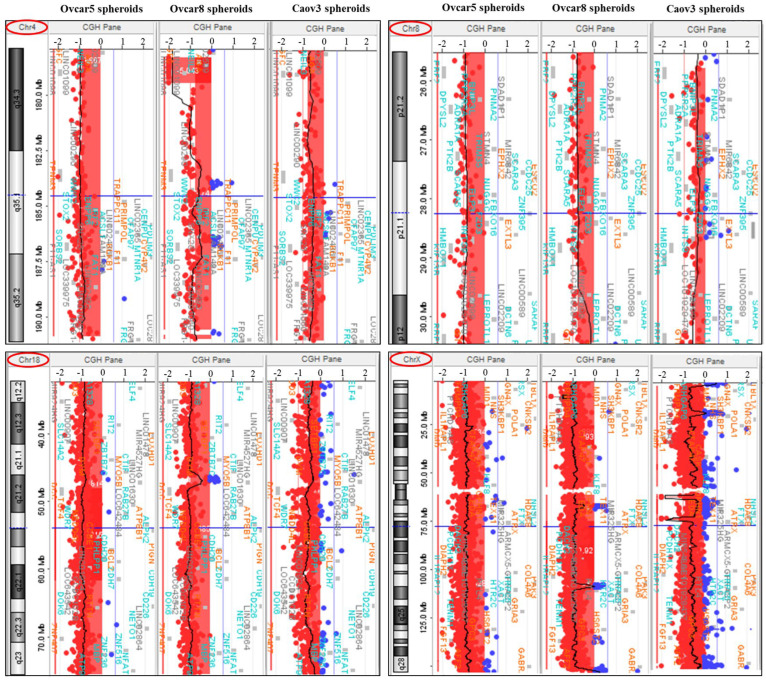
Copy number losses shared by all three CSCs subpopulations: 4q34.3-q35.2 (206 probes), 8p21.2-p21.1 (61 probes), 18q12.2-q23 (686 probes), and the entire chrX (2467 probes). Red: CN loss; blue: CN gain; red cycles: chromosome number.

Analyzing CNAs and SCAs shared by all the three CSCs subpopulations, we identified three autosomal regions: 4q34.3-q35.2 (nt 178341937-191133609, 206 probes), 8p21.2-p21.1 (nt 25774629-28393917, 61 probes), and 18q12.2-q23 (nt 32483714-75486904, 686 probes) (Figure 1). Moreover, loss of the entire chrX was observed (nt 4239811-152009316, 2467 probes).

**Figure 2:**
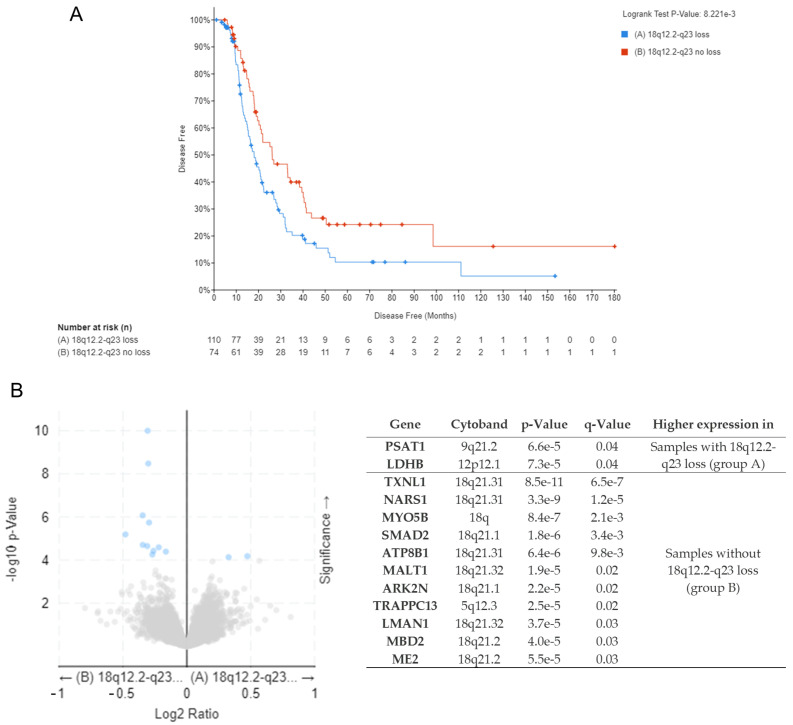
(**A**) Disease-free survival data of TCGA-OV patients divided into group A (samples with 18q12.2-q23 loss) and group B (samples without 18q12.2-q23 loss). (**B**) Differential expressed protein between samples of group A and group B analyzed using mass spectrometry (cBioPortal for Cancer Genomics web server). Blue dots: significant differentially expressed proteins between the two groups. Grey dots: not significant differentially expressed proteins between the two groups.

To evaluate the potential prognostic role of these copy number losses, we analyzed the correlation between loss in these regions and clinical parameters from the TCGA-OV (The Cancer Genome Atlas Ovarian Cancer) cohort using the cBioPortal for Cancer Genomics web server. Patients were divided into two groups based on the presence or absence of 18q12.2-q23 loss in tumor biopsies. The clinical data comparison between these two groups displayed a statistically significant difference in disease-free survival (DFS, Figure 2A). Moreover, an increased aneuploidy score and a higher fraction of genome altered in ovarian cancer samples carrying 18q12.2-q23 loss were observed (Table 1, Figure S1A,B). Thirteen proteins showed differential expression across the two groups of samples when protein levels were analyzed using mass spectrometry (Figure 2B). GO and pathway enrichment analyses on proteins overexpressed in the “no-loss” group of samples showed only one statistically significant GO term (negative regulation of transcription, DNA-templated). Enrichment analyses on two proteins overexpressed in the 18q “loss” group of samples identified a statistically significant increase in cysteine and methionine metabolism (KEGG pathway).

On the other hand, samples carrying 4q34.3-q35.2 loss showed an increase in the fraction of genome altered (*p* < 0.01), as well as in the mutational count (*p* < 0.01) (Table 1, Figure S1C,D). Finally, loss of 8p21.2-p21.1 was associated with a higher mutational count (*p* < 0.01) and increased tumor mutational burden (TMB) nonsynonymous (*p* < 0.01) (Table 1, Figure S1E,F). Taken together, these data suggest that the identified aberrations could correlate with generalized genetic–genomic instability.

As a result, we searched for potential variations in arm-level CNAs of other chromosomes in samples with the identified CN losses. We found statistically significant differences in the frequency of in arm-level CNAs in samples with CN losses with respect to samples without losses, especially in samples with 8p21.2-p21.1 loss (Table 1). This result, in addition to the previously reported higher tumor mutational burden, underlines that these samples are characterized by a great genetic instability.

Given the small number of genes in CN losses of chr8 and chr4, we carried out enrichment analyses in GO terms, REACTOME pathways, and KEGG pathways in order to uncover a possible explanation for the observed data. A statistically significant GO term (positive regulation of the apoptotic process, *p* < 0.01, BP: biological process) was found by DAVID analysis of the genes involved in 8p21.2-p21.1 CN, indicating that the loss of these genes may result in the loss of apoptosis. No interesting cluster was identified for 4q34.3-q35.2 genes.

### 3.3. Copy Number Losses Correlated with Clinical Parameters in the GDC Data Portal

We additionally investigated the potential correlations between clinical parameters and CN loss in 4q34.3-q35.2, 8p21.2-p21.1, and 18q12.2-q23 through the GDC Data Portal because the two repositories process the TCGA data differently, using different bioinformatics methods to analyze copy number alterations.

**Figure 3:**
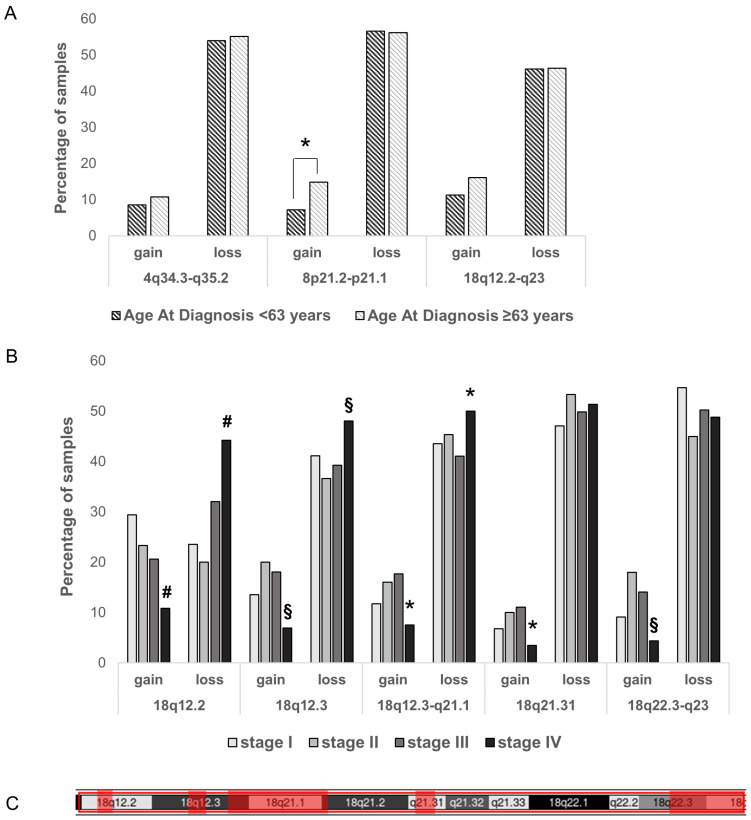
(**A**) 4q34.3-q35.2, 8p21.2-p21.1, and 18q12.2-q23 loss and gain percentages in samples of patients categorized by age at diagnosis. (**B**) Distribution of 18q copy number in samples categorized by clinical stage. #: statistically significant difference between stage IV samples and samples of all other stages, *p* < 0.05; §: statistically significant difference between stage IV and stage II/III samples, *p* < 0.05; *: statistically significant difference between stage IV and stage III samples, *p* < 0.05. (**C**) Localization of the 18q sub-regions represented in B (red boxes).

First, we divided the patients into two groups according to the median age at diagnosis (63 years) and checked the percentage of gains and losses in both groups (Figure 3A). A statistically significant difference (*p* < 0.05) in 8p21.2-p21.1 CN percentage was found; in particular, a lower percentage of 8p21.2-p21.1 gain was observed in samples of the youngest group (age at diagnosis under 63 years), while the percentage of CN loss was similar in both groups (Figure 3A).

On the other hand, considering the 18q12.2-q23, we evidenced a statistically significant enrichment in the percentage of stage IV samples with loss of this region (*p* < 0.05, Table S2). We checked the distribution of gain and loss also within the 18q12.2-q23 sub-regions (Figure 3B,C). Different percentages of CNAs among stages were identified (Table S2). In particular, stage IV showed a higher percentage of samples with loss of 18q12.2, as well as samples with loss of 18q12.3 and 18q12.3-q21.1 compared to other stages (Figure 3B,C). These findings could validate the role of this CN loss and demonstrate the involvement of 18q loss in ovarian cancer progression. No correlation between 8p or 4q losses and tumor stage was evidenced.

### 3.4. 18.q12.2 Region Correlated with Clinical Stage, Overall Survival, and Progression-Free Survival

**Figure 4:**
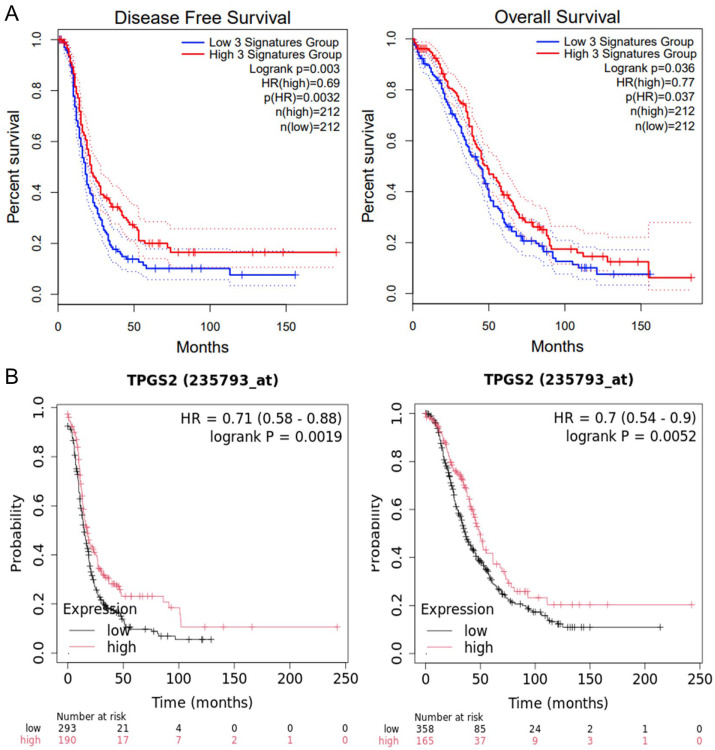
(**A**) GEPIA2 correlation between ovarian cancer patients’ DFS or OS and 18q12.2 genes (*FHOD3*, *TPGS2,* and *KIAA1328*, 3 signatures) expression (*p*-value < 0.05). Red line: high expression group; blue line: low expression group. (**B**) Kaplan–Meier Plotter correlation between patients’ DFS or OS and *TPGS2* expression (*p*-value < 0.01). Red line: high expression group; black line: low expression group.

Considering the previous results, we focused our attention on lost genes within the 18q12.2 region that showed a significant correlation (*p* < 0.05) with stage IV: *FHOD3*, *TPGS2*, and *KIAA1328* (Table S2). Using the GEPIA2 web server, we identified a statistically significant association between decreased expression of these genes and reduced disease-free survival (DFS) in ovarian cancer patients (*p* < 0.05), suggesting a prognostic relevance for this region. Moreover, we also found a link between gene expression and overall survival (OS) (Figure 4A, *p* < 0.05). The OncoDB web server further confirmed a correlation between the expression of these three genes and tumor stage, consistent with the observed CNA (Figure S2). Finally, to evaluate the role of each single gene, we consulted the Kaplan–Meier Plotter web server, selecting the serous histotype. Only *TPGS2A* showed a strong correlation between expression and both DFS and OS in ovarian cancer patients (Figure 4B), suggesting its pivotal role in ovarian cancer.

## 4. Discussion

Molecular biomarkers are defined as any measurable molecular indicator of risk disease or patient outcome. This category includes germline or somatic genetic variants, epigenetic signatures, transcriptional changes, and proteomic signatures [25]. Despite the vast number of investigations, the identification of novel biomarkers in ovarian cancer is still an urgent need [26].

The principal defining features of high-grade serous ovarian carcinoma (HGSOC), the most prevalent form of epithelial ovarian cancer, are copy number alterations and genomic rearrangements [27]. This makes genomic variants appealing as diagnostic or prognostic biomarkers, as well as for their easy detection [13].

Cancer stem cells (CSCs) are a subpopulation of cancer cells that are capable of self-renewal, differentiation, proliferation, and drug resistance. CSCs are also important in invasiveness and metastatic capability of tumors. For these reasons, it is understandable that a worse prognosis of the patient correlates with higher expression of the molecular signatures related to CSCs [28].

In a previous study, we isolated and characterized the CSC subpopulation from Caov3, Ovcar5, and Ovcar8 ovarian cancer cell lines. Caov3 and Ovcar8 are known to be representatives of HGSOC [19,20,22,29]. Concerning the Ovcar5 cell line, despite its origin still being debated, it is currently used as an HGSOC model [21,22], and for this reason, we decided to utilize three cell lines for our experiment. Remarkably, we identified a potential prognostic role of AhRR and PPP1R3C in serous ovarian cancer by bioinformatics analyses of array-CGH data performed on these CSCs [10]. In this work, we started from copy number losses shared by those CSC subpopulations in order to find chromosomal regions or genes that may be informative about patients’ prognosis.

First, we looked at the enriched pathways and GO terms of the 1253 genes that were found to be involved in copy number loss in all CSCs subpopulations. KEGG pathway analysis revealed a significant decrease in the mRNA surveillance pathway, including nonsense-mediated mRNA decay (NMD), nonstop mRNA decay (NSD), and no-go decay (NGD). NMD recognizes and degrades transcripts with a premature translation-termination codon, preventing the production of C-terminally truncated proteins that can have a deleterious effect in the cell [30]. Evidence has identified NMD as a key driver of tumorigenesis in a tumor-specific manner. In some cancers, NMD is enhanced to degrade certain mRNAs, including those encoding tumor suppressors. Conversely, in other tumors, NMD is inhibited, promoting the expression of oncoproteins or other proteins that support tumor growth and progression [31].

An interesting gene in this pathway was the *PPP2R2A* gene (the only one located at 8p21.1), which is deleted at high frequencies in luminal type B breast cancer and non-small cell lung cancer, as well as being one of the most common breakpoints in prostate cancer [32]. Loss of PPP2R2A inhibits homologous recombination DNA repair, suggesting it as a potential marker for PARP inhibitor responses in clinics [33].

REACTOME pathway enrichment analysis showed loss of downregulation of SMAD2/3:SMAD4 transcriptional activity. In the nucleus, the SMAD2/3:SMAD4 heterotrimer complex acts as a transcriptional regulator. SMAD2, SMAD3, and SMAD4 are considered to be key mediators of TGF-β signaling [34]. Ovarian tumors are significantly influenced by the TGF-β pathway and SMAD proteins [35]. In particular, upregulation of this pathway promotes the EMT process and enhances tumor cell resistance to paclitaxel [36]. Our results confirmed the important role of this pathway in ovarian CSCs.

GO enrichment analyses showed a statistically significant downregulation of gene silencing by miRNA in CSCs, together with a decrease of some GO terms related to mRNA processing. The maintenance of CSCs’ stemness and malignancy depends on mRNA modifications [37], and miRNA plays a significant role in this process. In fact, aberrant expression of miRNA, often due to genetic modifications, is essential for the initiation and progression of human cancers as they act as both tumor suppressors and oncogenes [38].

Taken together, these results validated our CSCs models, underlined some CSC characteristics, and confirmed the importance of copy number losses in tumorigenesis and cancer progression.

Subsequently, we identified only three CN losses and the loss of whole chromosome X shared by all spheroids and investigated a possible correlation with patients’ prognosis suggested by their presence in all CSCs models. Loss of chromosome X was abundantly reported in cancer as a potential mechanism of X-linked tumor suppressor gene inactivation, so we focused our attention on the other three CN losses [39,40].

Loss of 4q34.3-q35.2 correlated with an increase in the fraction of genome altered, as well as in the mutational count in these samples, suggesting an increased genomic instability. Terminal 4q loss has been found in colorectal cancer as a marker of advanced stage [41], in hepatoblastoma as a poor prognostic factor [42], and in intrahepatic cholangiocarcinoma associated with a high histological grade [43]. Moreover, deletion of 4q34.3 predicted early relapse after adjuvant chemotherapy in lung adenocarcinoma [44]. Frequent LOH (loss of heterozygosity) in the 4q terminal region in hepatocellular carcinoma, head and neck squamous cell carcinoma, and oral carcinoma has also been reported [42]. With regard to ovarian cancer, a loss of 4q34.3 in mucinous and clear cell ovarian cancer cell lines [12] and a potential correlation between 4q35.2 loss and chemoresistance [45] were previously reported.

Loss of 8p21.2-p21.1 in ovarian cancer biopsies was associated with a higher mutational count and increased TMB (nonsynonymous) together with a statistically significant increase in arm-level CNAs, indicating a great genetic instability of these samples. Bioinformatics analyses of the genes involved in 8p21.2-p21.1 CNA revealed a statistically significant cluster in the positive regulation of the apoptotic process, suggesting that the loss of these genes may lead to the loss of apoptosis.

Frequent deletion (23%) of 8p21.2 was identified in TCGA tumors and reported in previous studies for ovarian cancer, particularly for serous histology and high-grade and chemoresistant samples [46]. Another study identified loss on 13q32.1 and 8p21.1 as the most reliable combination for detecting chemoresistant disease, with *EXTL3* as a potential gene linked to antineoplastic drug-resistance [45].

Kaveh et al. analyzed copy number data from breast, ovarian, endometrial, and cervical cancers and identified 8p21.2 loss in cancers of the reproductive system, indicating *BNIP3L* (a proapoptotic gene) and *PPP2R2A* as interesting tumor suppressor genes [47]. Our results support *PPP2R2A*’s role in ovarian CSCs also.

Moreover, we identified for the first time a statistically significant difference in 8p21.2-p21.1 gain between early- and late-onset ovarian cancer (cut-off 63 years), suggesting a potential favorable prognostic role of this gain, in accordance with data observed in samples with loss.

As shown for loss of 4q34.3-q35.2 and 8p21.2-p21.1, 18q12.2-q23 loss correlated with clinical parameters related to genomic instability, such as an increased aneuploidy score and arm-level CNAs, as well as a higher fraction of altered genome. Intriguingly, a statistically significant difference in disease-free survival of patients with or without loss was found. Pathway enrichment analyses of differentially expressed proteins in the two groups revealed a statistically significant increase in cysteine and methionine metabolism in the 18q loss group (PSAT1 and LDHB proteins).

Phosphoserine aminotransferase 1 (PSAT1) catalyzes the second step of the serine-glycine biosynthesis pathway, and its overexpression was reported in ovarian cancer [48], lung adenocarcinoma [49], and breast cancer [50]. Lactate dehydrogenase (LDH) plays key roles in cancer metabolism reprogramming [51]. LDHA directly catalyzes the conversion of pyruvate to lactate; on the contrary, LDH-B converts lactate to pyruvate. Upregulation of LDH-B in tumors has been reported and correlated with disease progression and poor prognosis [50,51,52]. These data could explain the reduced DFS of patients with 18q loss.

Additionally, a link between 18q12.2-q23 and the stage of the tumor was found; in fact, a statistically significant increase of samples with CN loss in this region was found in stage IV. Interestingly, within the 18q12.2-q23 region, different sub-regions with different percentages of alterations among stages were present. In particular, the percentage of CN loss of the 18q12.2 region (containing *FHOD3*, *TPGS2*, and *KIAA1328*) was significantly increased in stage IV samples with respect to all other samples. Genomic observation was supported by mRNA data from the OncoDB web server that confirmed a correlation between the expression of these three genes and the clinical stage of the tumor. Moreover, a strong correlation between *TPGS2A* expression and both DFS and OS in ovarian cancer patients could suggest for the first time its prognostic role in ovarian cancer.

## 5. Conclusions

In conclusion, we analyzed copy number losses shared by our previously isolated and characterized ovarian CSC subpopulations. Pathway and gene ontology further validated our CSC models, underlining some CSC characteristics and confirming the importance of copy number losses in tumorigenesis and cancer progression.

Then, starting from these CN losses, we validated their potential prognostic relevance by analyzing the TCGA cohort. Our analysis of copy number alterations in ovarian CSCs revealed novel insights by identifying three specific copy number losses associated with higher genetic instability and patients’ prognosis. The identified potential candidate genes not only enhance our understanding of the role of numerical aberrations as biomarkers but also suggest new avenues for targeted therapies. By elucidating the pathways influenced by these genetic alterations, our findings could help the development of personalized treatment strategies, ultimately aiming to improve patient outcomes and more effectively manage cancer progression.

## Figures and Tables

**Table 1:** Variations in clinical parameters and chromosome arm-level CNAs in samples carrying 4q34.3-q35.2, 8p21.2-p21.1, or 18q12.2-q23 loss. Only chromosome arms with statistically significant differences are reported.

	4q34.3-q35.2 Loss vs. No Loss	8p21.2-p21.1 Loss vs. No Loss	18q12.2-q23 Loss vs. No Loss
Aneuploidy score	unvaried	unvaried	↑
Fraction of genome altered	↑	unvaried	↑
Mutational count	↑	↑	unvaried
TMB (nonsynonymous)	unvaried	↑	unvaried
2p	unvaried	unvaried	↓ CN gain
2q	unvaried	↑ CN gain	↓ CN gain
3q	unvaried	↑ CN gain	unvaried
4p	↑ CN loss	unvaried	↑ CN loss
4q	**arm involved in CNA**	unvaried	↑ CN loss
5p	unvaried	↑ CN gain	unvaried
5q	unvaried	↑ CN loss	unvaried
6p	unvaried	↑ CN gain	↑ CN loss
7p	unvaried	unvaried	↑ CN loss
7q	unvaried	unvaried	↑ CN loss
8p	unvaried	**arm involved in CNA**	unvaried
9p	unvaried	unvaried	↑ CN loss
9q	↑ CN loss	↑ CN loss	unvaried
10p	unvaried	↑ CN gain	unvaried
10q	unvaried	↑ CN loss	unvaried
11q	unvaried	↑ CN gain	↑ CN loss
12q	unvaried	unvaried	↑ CN loss
13q	unvaried	↑ CN loss	unvaried
14q	unvaried	↑ CN loss	unvaried
15q	unvaried	↑ CN loss	unvaried
16p	↑ CN loss	unvaried	↑ CN loss
16q	unvaried	↑ CN loss	↑ CN loss
17p	unvaried	↑ CN gain	unvaried
18p	unvaried	↑ CN gain	↑ CN loss
18q	↑ CN loss	unvaried	**arm involved in CNA**
19p	unvaried	unvaried	↑ CN loss
20p	↑ CN gain	unvaried	unvaried
20q	↑ CN gain	↑ CN gain	unvaried

↑: increase; ↓: decrease.

## Data Availability

The data that support the findings of this study are available from the corresponding author [D.C.] upon reasonable request.

## Supplementary Materials

The following supporting information can be downloaded at https://www.mdpi.com/article/10.3390/genes15111487/s1. Table S1: KEGG pathway, REACTOME pathway, and GO (BP: biological process, and MF: molecular function) enrichment analyses of 1253 genes involved in copy number losses. Figure S1: Correlation between copy number losses and clinical parameters. Table S2: 18q12.2-q23 loss in different tumor stages. Figure S2: OncoDB correlation between expressions of FHOD3, TPGS2, and KIAA1328 and the clinical stage of a tumor.
