# Supplementary material for: Prognostic Relevance of Copy Number Losses in Ovarian Cancer
Source: Genes (Basel). 2024 Nov 19;15(11):1487. doi: 10.3390/genes15111487 (PMC11593593; doi:10.3390/genes15111487)
Supplement: Supplementary file 1 [file genes-15-01487-s001.zip › Table S1.pdf]

Table S1: KEGG pathway, REACTOME pathway and GO term (BP: biological process and MF: molecular function) enrichment analyses of 1253 genes involved in copy number losses.

Category	Term	Count	P-value
KEGG	mRNA surveillance pathway	12	6,6E-4
	AGE-RAGE signaling pathway in diabetic complications	9	2,7E-2
	Arginine and proline metabolism	6	3,2E-2
	NF-kappa B signaling pathway	9	3,3E-2
	Neuroactive ligand-receptor interaction	21	3,9E-2
	Histidine metabolism	4	4,2E-2
REACTOME	Downregulation of SMAD2/3:SMAD4 transcriptional activity	6	3,3E-3
	Protein-protein interactions at synapses	9	9,2E-3
	RAB geranylgeranylation	7	2,4E-2
	Receptor-type tyrosine-protein phosphatases	4	3,0E-2
	Transcriptional activity of SMAD2/SMAD3:SMAD4 heterotrimer	6	3,0E-2
	RAC3 GTPase cycle	8	4,2E-2
	RHOU GTPase cycle	5	4,7E-2
GOTERM_BP	gene silencing by miRNA	60	1,0E-10
	negative regulation of endopeptidase activity	12	5,2E-6
	negative regulation of transcription from RNA polymerase II promoter	72	5,6E-6
	poly(A)+ mRNA export from nucleus	6	3,0E-3
	regulation of transcription, DNA-templated	57	5,9E-3
	negative regulation of vascular smooth muscle cell proliferation	7	7,6E-3
	gamete generation	5	7,9E-3
	negative regulation of protein ubiquitination	8	9,1E-3
	regulation of presynapse assembly	6	1,0E-2
	cellular response to hypoxia	13	1,2E-2
	organ growth	5	1,3E-2
	snRNA 3'-end processing	4	1,4E-2
	brain development	16	1,6E-2
	negative regulation of cell adhesion molecule production	4	1,8E-2
	negative regulation of protein acetylation	3	2,2E-2
	negative regulation of vascular associated smooth muscle cell migration	5	2,3E-2
	regulation of transmembrane transporter activity	3	3,0E-2
	synapse organization	7	3,5E-2
	neuron differentiation	13	3,7E-2
	negative regulation of autophagy	7	3,8E-2
	negative regulation of angiogenesis	11	3,9E-2
	negative regulation of G0 to G1 transition	3	4,0E-2
	negative regulation of ERK1 and ERK2 cascade	8	4,0E-2
	cellular response to amino acid stimulus	7	4,3E-2
	mRNA export from nucleus	7	4,3E-2

GOTERM_MF	mRNA binding involved in posttranscriptional gene silencing	31	5,6E-6
	histone deacetylase binding	17	8,2E-5
	serine-type endopeptidase inhibitor activity	15	1,4E-4
	methylated histone binding	12	2,4E-4
	mRNA 3'-UTR binding	22	1,5E-3
	neurexin family protein binding	5	2,7E-3
	poly(A) binding	6	7,1E-3
	protein binding	555	9,2E-3
	carbon-sulfur lyase activity	3	9,6E-3
	mRNA binding	18	2,6E-2
	chromatin binding	30	3,1E-2
	transferase activity, transferring acyl groups other than amino-acyl groups	4	4,1E-2
	RNA polymerase II sequence-specific DNA binding transcription factor binding	14	4,3E-2
