# Supplementary material for: Prognostic Relevance of Copy Number Losses in Ovarian Cancer
Source: Genes (Basel). 2024 Nov 19;15(11):1487. doi: 10.3390/genes15111487 (PMC11593593; doi:10.3390/genes15111487)
Supplement: Supplementary file 1 [file genes-15-01487-s001.zip › Table S2.pdf]

Genes	Chr	stage I		stage II		stage III		stage IV	
		gain_cases	loss_cases	gain_cases	loss_cases	gain_cases	loss_cases	gain_cases	loss_cases
**FHOD3**	18q12.2	5	4	7	6	93	139	10	44 #§°
**TPGS2**	18q12.2	5	4	7	6	91	142	9	39 #§°
**KIAA1328**	18q12.2	5	4	7	6	89	143	9	31 #§
**CELF4**	18q12.2	5	5	7	6	90	141	12	29
**PIK3C3**	18q12.3	3	7	6	11	80	160	5	35 §°
**RIT2**	18q12.3	2	7	6	11	81	164	6	43 §°
**SYT4**	18q12.3	2	7	6	11	78	165	7	46 §°
**SETBP1**	18q12.3	2	7	5	12	78	170	8	43 °
**SLC14A2**	18q12.3	2	7	5	13	78	171	8	44 °
**SLC14A1**	18q12.3	2	7	5	13	78	169	9	43 °
**SIGLEC15**	18q12.3	2	7	5	13	78	171	9	41
**EPG5**	18q12.3, 18q21.1	2	7	5	13	78	171	9	45 °
**PSTPIP2**	18q21.1	2	7	5	13	78	172	9	42
**ATP5F1A**	18q21.1	2	7	5	13	78	171	9	43 °
**HAUS1**	18q21.1	2	7	5	13	79	171	9	46 °
**C18orf25**	18q21.1	2	7	5	13	78	170	8	43 °
**RNF165**	18q21.1	2	7	5	13	77	172	8	46 °
**LOXHD1**	18q21.1	2	7	5	13	74	176	8	40
**ST8SIA5**	18q21.1	2	7	5	13	74	176	9	44
**PIAS2**	18q21.1	2	7	5	13	75	175	7	36
**KATNAL2**	18q21.1	2	7	5	13	74	177	7	40 °
**ELOA3D**	18q21.1	2	7	5	13	74	177	7	44 °
**ELOA3C**	18q21.1	2	7	5	13	74	177	7	43 °
**ELOA3B**	18q21.1	2	7	5	13	74	177	7	43 °
**ELOA3**	18q21.1	2	7	5	13	74	177	7	43 °
**ELOA2**	18q21.1	2	7	5	13	74	177	7	44 °
**HDHD2**	18q21.1	2	7	5	13	73	177	6	37 °
**IER3IP1**	18q21.1	2	7	5	13	73	177	6	43 °

		stage I		stage II		stage III		stage IV	
		gain_cases	loss_cases	gain_cases	loss_cases	gain_cases	loss_cases	gain_cases	loss_cases
**SKOR2**	18q21.1	2	7	5	13	73	177	6	46 °
**SMAD2**	18q21.1	2	8	5	13	73	182	6	43 °
**ZBTB7C**	18q21.1	2	8	4	14	69	184	5	46 °
**CTIF**	18q21.1	2	8	5	14	71	183	5	45 °
**SMAD7**	18q21.1	2	8	5	14	70	183	5	46 °
**DYM**	18q21.1	2	8	5	14	66	191	5	44 °
**C18orf32**	18q21.1	2	8	5	14	65	191	5	38 °
**RPL17-C18orf32**	18q21.1	2	8	5	14	65	191	5	44 °
**RPL17**	18q21.1	2	8	5	14	65	191	5	44 °
**LIPG**	18q21.1	2	8	5	14	64	192	5	36
**ACAA2**	18q21.1	2	8	5	14	63	195	5	43 °
**MYO5B**	18q21.1	2	8	4	16	62	196	4	45 °
**CFAP53**	18q21.1	2	8	4	16	62	199	4	46 °
**MBD1**	18q21.1	2	8	4	16	62	198	4	42 °
**CXXC1**	18q21.1	2	8	4	16	62	199	4	43 °
**SKA1**	18q21.1	2	8	4	16	62	199	5	46 °
**MAPK4**	18q21.1, 18q21.2	2	8	4	16	59	198	5	43
**MRO**	18q21.2	2	8	4	16	59	199	5	43
**ME2**	18q21.2	2	8	4	16	60	198	5	46 °
**ELAC1**	18q21.2	2	8	4	16	60	198	5	39
**SMAD4**	18q21.2	2	8	4	16	60	197	5	40
**MEX3C**	18q21.2	2	8	4	16	60	197	5	46 °
**DCC**	18q21.2	2	8	3	16	57	204	5	43
**MBD2**	18q21.2	2	8	3	15	56	210	6	43
**POLI**	18q21.2	2	8	3	15	56	210	6	36
**STARD6**	18q21.2	2	8	3	15	56	210	6	44
**C18orf54**	18q21.2	2	8	3	15	56	210	6	44

		stage I		stage II		stage III		stage IV	
		gain_cases	loss_cases	gain_cases	loss_cases	gain_cases	loss_cases	gain_cases	loss_cases
**DYNAP**	18q21.2	2	8	4	15	55	210	6	46
**RAB27B**	18q21.2	2	8	4	15	55	210	5	46
**CCDC68**	18q21.2	2	8	4	15	55	212	5	43
**TCF4**	18q21.2	2	8	3	16	53	214	4	44
**TXNL1**	18q21.31	2	8	3	16	49	217	3	40
**WDR7**	18q21.31	2	8	3	16	49	218	3	43 °
**BOD1L2**	18q21.31	1	8	3	16	50	219	3	43 °
**ST8SIA3**	18q21.31	1	8	3	16	49	220	3	45 °
**ONECUT2**	18q21.31	1	8	3	16	50	220	3	46 °
**FECH**	18q21.31	1	8	3	16	51	220	3	42 °
**ATP8B1**	18q21.31	1	8	3	16	49	222	3	46 °
**NEDD4L**	18q21.31	1	8	4	15	48	223	4	31
**ALPK2**	18q21.31, 18q21.32	1	8	4	15	52	223	4	31
**MALT1**	18q21.32	1	8	4	15	50	223	4	43
**ZNF532**	18q21.32	1	8	4	15	50	222	5	39
**SEC11C**	18q21.32	1	8	4	15	50	221	5	40
**GRP**	18q21.32	1	8	4	15	50	221	5	46
**RAX**	18q21.32	1	8	4	15	50	221	5	44
**CPLX4**	18q21.32	1	8	4	15	50	222	5	45
**LMAN1**	18q21.32	1	8	4	15	50	222	5	37
**CCBE1**	18q21.32	1	8	4	15	52	221	5	39
**PMAIP1**	18q21.32	1	7	4	15	52	222	5	35
**MC4R**	18q21.32	1	7	4	15	50	221	5	44
**CDH20**	18q21.33	1	8	4	15	54	218	5	43
**RNF152**	18q21.33	1	8	4	15	56	216	5	43
**PIGN**	18q21.33	1	8	4	15	57	216	5	35
**RELCH**	18q21.33	2	8	5	15	59	219	5	43
**TNFRSF11A**	18q21.33	2	8	5	15	58	218	5	44

		stage I		stage II		stage III		stage IV	
		gain_cases	loss_cases	gain_cases	loss_cases	gain_cases	loss_cases	gain_cases	loss_cases
**ZCCHC2**	18q21.33	2	8	5	15	62	216	5	41
**PHLPP1**	18q21.33	2	8	5	15	59	216	5	37
**BCL2**	18q21.33	2	8	5	15	60	215	6	46
**KDSR**	18q21.33	2	8	5	15	61	216	5	43
**VPS4B**	18q21.33	2	8	5	15	61	216	5	45
**SERPINB5**	18q21.33	2	8	5	15	61	216	5	44
**SERPINB12**	18q21.33	2	8	5	15	61	217	5	44
**SERPINB13**	18q21.33	2	8	5	15	60	217	5	43
**SERPINB4**	18q21.33	2	8	5	15	59	217	5	40
**SERPINB3**	18q21.33	2	8	5	15	59	217	5	42
**SERPINB11**	18q21.33	2	8	5	15	59	218	5	41
**SERPINB7**	18q21.33	2	8	5	15	58	220	5	43
**SERPINB2**	18q21.33, 18q22.1	2	8	4	15	58	222	4	42
**SERPINB10**	18q21.33, 18q22.1	2	8	4	15	58	220	4	42
**HMSD**	18q22.1	2	8	4	15	58	220	4	37
**SERPINB8**	18q22.1	2	8	4	15	58	220	4	44 °
**CDH7**	18q22.1	2	9	3	15	62	219	5	45
**CDH19**	18q22.1	2	9	3	15	64	216	5	40
**DSEL**	18q22.1	2	9	3	14	64	217	5	44 °
**TMX3**	18q22.1	4	9	4	14	60	220	5	44
**CCDC102B**	18q22.1	3	9	4	14	60	219	6	41
**DOK6**	18q22.2	2	9	4	14	59	223	5	44
**CD226**	18q22.2	2	9	4	14	58	222	5	45
**RTTN**	18q22.2	2	9	4	14	57	223	5	42
**SOCS6**	18q22.2	2	9	4	14	59	223	5	45
**CBLN2**	18q22.3	2	9	6	14	62	222	4	45 §°
**NETO1**	18q22.3	2	9	6	14	62	222	5	43 §

		stage I		stage II		stage III		stage IV	
		gain_cases	loss_cases	gain_cases	loss_cases	gain_cases	loss_cases	gain_cases	loss_cases
**FBXO15**	18q22.3	2	9	6	14	62	221	4	38 §
**TIMM21**	18q22.3	2	9	6	14	63	221	4	44 °
**CYB5A**	18q22.3	2	9	6	13	63	221	4	46 §°
**C18orf63**	18q22.3	2	9	6	13	62	221	4	45 §°
**DIPK1C**	18q22.3	2	9	6	13	61	220	4	43 §°
**CNDP2**	18q22.3	2	9	6	13	61	220	4	44 §°
**CNDP1**	18q22.3	2	9	6	13	61	220	4	45 §°
**ZNF407**	18q22.3	2	9	6	14	62	221	4	39 §
**ZADH2**	18q22.3	1	10	5	14	62	221	4	45 §°
**TSHZ1**	18q22.3	1	10	5	14	62	220	4	41 °
**SMIM21**	18q23	1	10	5	14	62	220	4	36
**ZNF516**	18q23	1	10	4	14	64	222	4	39 °
**ZNF236**	18q23	1	10	4	14	63	221	3	39 °
**MBP**	18q23	1	10	4	13	63	222	3	44 °
**GALR1**	18q23	2	9	4	13	63	226	3	44 °
**SALL3**	18q23	1	9	5	13	61	223	3	37 §°
**ATP9B**	18q23	1	9	6	13	59	224	3	39 §°
**NFATC1**	18q23	1	9	6	13	61	222	3	44 §°

#p<0.05 stage IV versus stage I, §p<0.05 stage IV versus stage II, °p<0.05 stage IV versus stage III
