# Supplementary figures and images for: Prognostic Relevance of Copy Number Losses in Ovarian Cancer
Source: Genes (Basel). 2024 Nov 19;15(11):1487. doi: 10.3390/genes15111487 (PMC11593593; doi:10.3390/genes15111487)

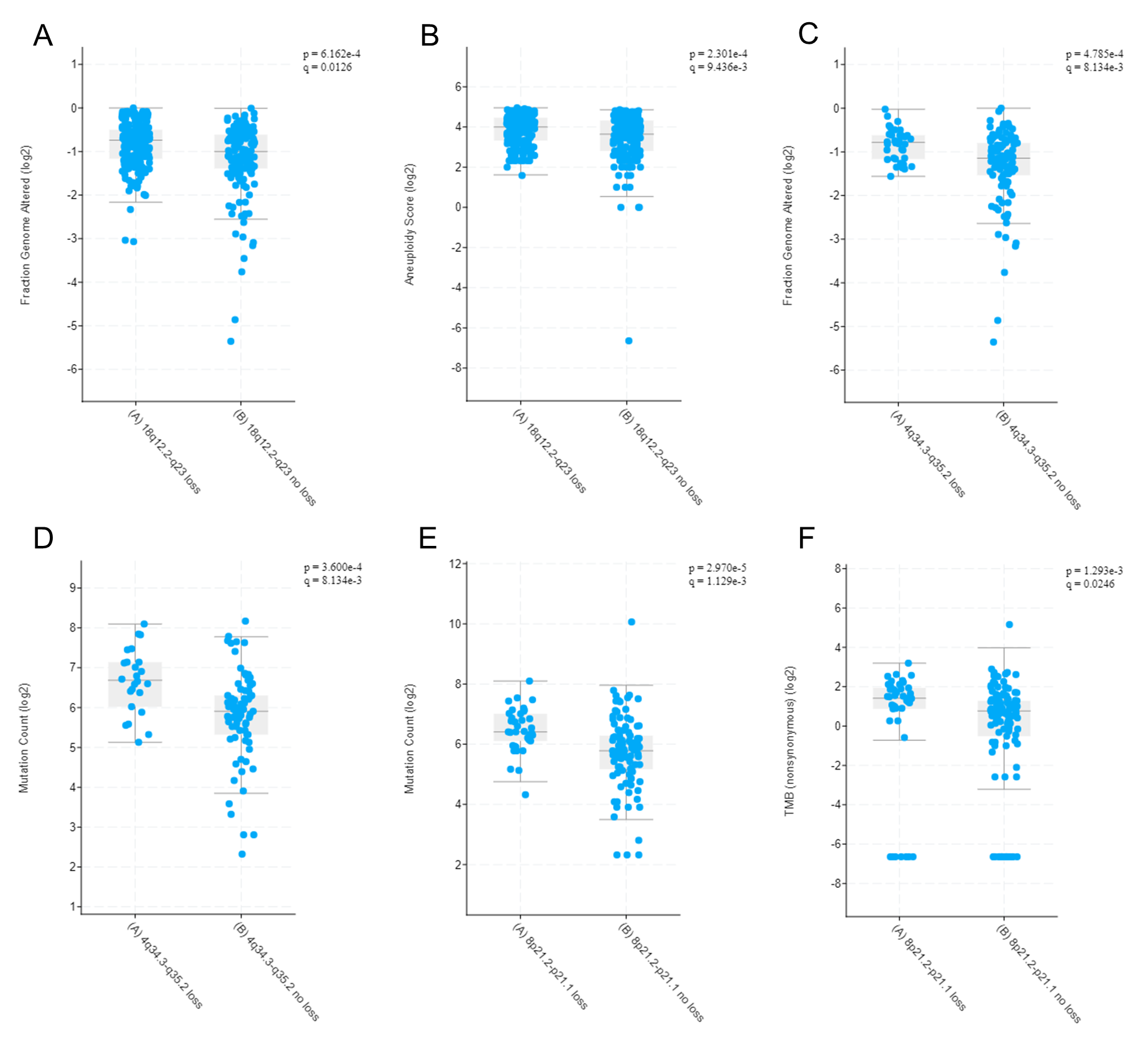

Supplement: Supplementary file 1 [file genes-15-01487-s001.zip › Figure S1.tif]

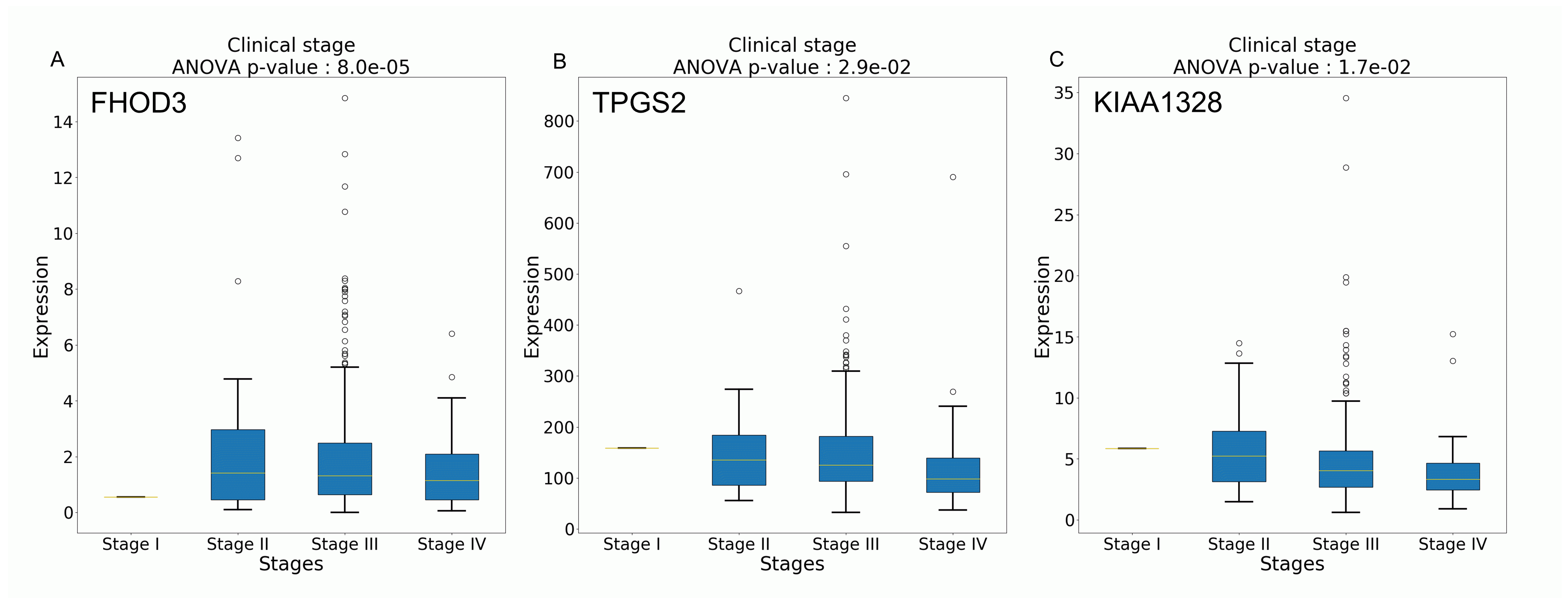

Supplement: Supplementary file 1 [file genes-15-01487-s001.zip › Figure S2.tif]
